# Interictal Epileptiform Discharge Dynamics in Peri-sylvian Polymicrogyria Using EEG-fMRI

**DOI:** 10.3389/fneur.2021.658239

**Published:** 2021-06-03

**Authors:** Noa Cohen, Yoram Ebrahimi, Mordekhay Medvedovsky, Guy Gurevitch, Orna Aizenstein, Talma Hendler, Firas Fahoum, Tomer Gazit

**Affiliations:** ^1^Sagol Brain Institute, Wohl Institute for Advanced Imaging, Sourasky Medical Center, Tel Aviv, Israel; ^2^Sackler School of Medicine, Tel Aviv University, Tel Aviv, Israel; ^3^Department of Neurology, Agnes Ginges Center of Neurogenetics, Hadassah-Hebrew University Medical Center, Jerusalem, Israel; ^4^Department of Diagnostic Imaging, Sourasky Medical Center, Tel Aviv, Israel; ^5^School of Psychological Science, Tel Aviv University, Tel Aviv, Israel; ^6^Sagol School of Neuroscience, Tel Aviv University, Tel Aviv, Israel; ^7^Electroencephalography and Epilepsy Unit, Sourasky Medical Center, Tel Aviv, Israel

**Keywords:** epilepsy, interictal epileptiform discharges, polymicrogyria, EEG-fMRI, interictal dynamics

## Abstract

Polymicrogyria (PMG) is a common malformation of cortical development associated with a higher susceptibility to epileptic seizures. Seizures secondary to PMG are characterized by difficult-to-localize cerebral sources due to the complex and widespread lesion structure. Tracing the dynamics of interictal epileptiform discharges (IEDs) in patients with epilepsy has been shown to reveal the location of epileptic activity sources, crucial for successful treatment in cases of focal drug-resistant epilepsy. In this case series IED dynamics were evaluated with simultaneous EEG-fMRI recordings in four patients with unilateral peri-sylvian polymicrogyria (PSPMG) by tracking BOLD activations over time: before, during and following IED appearance on scalp EEG. In all cases, focal BOLD activations within the lesion itself preceded the activity associated with the time of IED appearance on EEG, which showed stronger and more widespread activations. We therefore propose that early hemodynamic activity corresponding to IEDs may hold important localizing information potentially leading to the cerebral sources of epileptic activity. IEDs are suggested to develop within a small area in the PSPMG lesion with structural properties obscuring the appearance of their electric field on the scalp and only later engage widespread structures which allow the production of large currents which are recognized as IEDs on EEG.

## Introduction

Recent findings point to both ictal (during seizures) and interictal (between seizures) epileptic event types as arising from a common cortical source, termed the epileptogenic zone [EZ; (1, 2)]. Though the precise relationship between these epileptic phenomena and their cerebral origins is still not sufficiently understood (3, 4), interictal activity is often utilized in order to identify the generators of seizure activity. This is particularly relevant for patients with drug-resistant epilepsy for which achieving seizure control highly depends on correct EZ localization and its removal by surgical resection. Such patients undergo extensive pre-surgical testing including neuroimaging, electrophysiology and neuropsychological examinations for identifying their seizure sources. In most cases, no single method is sufficient for reliably assessing this area's location and extent and even after exhaustive testing, many surgical procedures are unsuccessful (5, 6). When possible, seizure semiology and neurophysiology are used to study the sources of seizure activity, however in many cases seizures are difficult to capture in a clinical setting. Interictal activity, however, may be captured non-invasively and in an outpatient setting, providing more accessible options for EZ identification and adding crucial localizing information (7).

PMG is a common malformation of cortical development, characterized by an excessively folded cortical ribbon of miniature, individually thin convolutions (8). PMG, including the most common peri-sylvian (PSPMG) subtype, has been associated with a wide range of clinical manifestations such as cognitive impairment (9), focal neurological deficits (10) and intractable epilepsy (11). Contradicting findings have been reported regarding the areas generating ictal and interictal events in patients with PMG, which include areas within the PMG (12) and outside of it (13). In the latter study, Jacobs and colleagues suggested that aberrant synaptic connectivity develops around the microgyri and produces a focal epileptogenic zone whose capacity to generate epileptiform activity does not depend on connections with the malformation itself. Intralesional recordings in humans with PMG demonstrated a large epileptic network involving both the lesion and non-malformed cortex (14), while intracranial electroencephalography (iEEG) studies have shown that the seizure onset zone may only partially overlap with the PMG cortex (15). Surgical resection of sub-portions of the malformed cortex have been shown to result in positive surgical outcomes (16, 17), suggesting that a region within the PMG may be the source of abnormal electrical activity.

In many cases, fast propagation and dynamic distribution of IEDs confound the localization of their cerebral sources (18, 19), leading to misinterpretation of pre-surgical testing and mis-localization of the EZ. Using high resolution imaging methods, studies have shown that source localization may be improved when focusing on the early phases of the interictal discharge, as the later stages are associated with more widespread activity (1, 18). The simultaneous measurement of electroencephalography (EEG) and functional magnetic resonance imaging (fMRI) is a non-invasive neuroimaging method which provides valuable information concerning the localization of regions generating IEDs (20, 21). It is a useful diagnostic tool to guide pre-surgical evaluation of refractory epilepsy, assisting in depth electrode implantation (22) and consistent with epileptogenic tissue (23). EEG-fMRI had also been successfully used for localizing the EZ in patients with cortical and subcortical malformations such as focal cortical dysplasia (24), cortical tubers (25) and polymicrogyria [PMG; (26)]. Notwithstanding, the efficacy of this method is still under investigation and its sensitivity to epileptic sources is reportedly equal to or lower than other diagnostic methods (27). Recent work suggests the sensitivity of EEG-fMRI may be enhanced by taking into account the temporal dynamics of epileptic activity (28). Epileptogenic lesions such as PMG may affect IED dynamics in predictable ways (29), allowing to test the ability of EEG-fMRI to map IED pathways over time.

IED dynamics in PSPMG have been studied in animal models but are difficult to assess in humans non-invasively. This may be due to source epileptic structures being located deep in the brain in unorganized cortical structures not able to produce sufficiently strong dipoles as to be observed from the scalp (30). Thus, it is not always clear whether the fields observed by scalp EEG and MEG are related to the source of the activity or due to its spread to neighboring superficial and more organized cortical structures. The purpose of the current study was to examine the dynamics of IEDs as reflected in EEG-fMRI BOLD activations correlated with observed IEDs on the scalp of patients with PSPMG. We hypothesized that IEDs observed on the scalp would correspond to IED spread to superficial cortical structures while BOLD activity within the PMG could be observed in the time preceding IED appearance on scalp recordings and reveal their focal sources.

## Methods

### Patients

Included in this study are all four patients diagnosed with PSPMG and refractory epileptic seizures which underwent an EEG-fMRI scan at the Tel Aviv Sourasky Medical Center between March 2014 and June 2019. Patients' clinical and imaging findings are detailed below and summarized in Table 1. Neuroimaging results include scalp EEG seizure onset, main MRI finding, major dipole concentration on MEG, significant PET results and peak BOLD activations observed during EEG-fMRI. Activations are reported at two time points: 2.25 s after IED appearance on scalp EEG, corresponding to pre-IED activity; and 5.25 s after IED appearance on scalp EEG, corresponding to IED initiation according to the standard hemodynamic response function [HRF; (31)].

**Table 1:** Summary of patient details and main neuroimaging findings.

**Patient**	**Age (years)**	**Onset (years)**	**Hand-edness**	**Ictal EEG onset**	**MRI**	**MEG**	**PET**	**Pre-IED peak activity**	**IED peak activity**
1	24	13	R	R FT	R PSPMG	R TP	Diff (HM)	R In	R IFG R In
2	19	10	R	R F	R PSPMG	R FT	L T (HM)	R SFG R Op	R SFG
3	26	16	R	R FT	R PSPMG	R T	R TPO L T (HM)	R In^*^	R MTG R STG
4	40	19^**^	R	R T	R PSPMG R Schize- ncephaly	–	RT (HM)	R TOp	R TOp

*R, right; L, left; PSPMG, perisylvian polymicrogyria; T, temporal; O, occipital; P, parietal; F, frontal; In, insula; Op, operculum; IFG, inferior frontal gyrus; SFG, superior frontal gyrus; MTG, medial temporal gyrus; STG, superior temporal gyrus; HM, hypometabolism; Diff, diffuse.*

* *Not significant after FWE correction.*

** *Onset age of typical seizures. Patient reported childhood absence seizures as well.*

Patient 1 is a right-handed female aged 24 suffering from epilepsy since the age of 13 involving frequent complex partial seizures. Ictal EEG shows right frontotemporal onset with fast contralateral spread. Neurological deficits include memory loss and difficulties with word retrieval, concentration and motivation. MRI shows a right PSPMG with pre-central and post-central involvement and right hemispheric atrophy. Interictal PET scan showed diffuse hypometabolism. Interictal MEG sources were found in right temporo-parietal areas.

Patient 2 is a right-handed female aged 19 suffering from epilepsy since the age of 10. Ictal semiology is concordant with right hemisphere onset with or without secondary generalization. She suffers from left sided hemiparesis from childhood. MRI shows diffuse right PSPMG including pre and post central areas with loss of volume in the right hemisphere. PET shows right temporo-parietal hypometabolism and left temporal hypometabolism. MEG shows right fronto-temporal dipoles.

Patient 3 is a right-handed female aged 26 suffering from epilepsy since the age of 16. She has a mild left hemiparesis and focal seizures originating from the right antero-mid temporal areas. Right PSPMG is seen on MRI with loss of volume in the right hemisphere and specifically in right hippocampus. MEG reveals right temporal dipole concentration and PET shows right temporo-parieto-occipital and left temporal hypometabolism.

Patient 4 is a right-handed female aged 42 suffering from early onset epilepsy which subsided for several years. At the age of 19 seizures reappeared characterized as focal seizures with impaired consciousness accompanied by right temporal sharp and slow waves on EEG. MRI shows right sided PSPMG and schizencephaly, and a PET scan points to right lateral temporal hypometabolism.

### Data Acquisition

The study was approved by the Tel-Aviv Sourasky Medical Center Ethical Review Board. Written informed consent for participation in the study was obtained from all patients.

**Figure 1:**
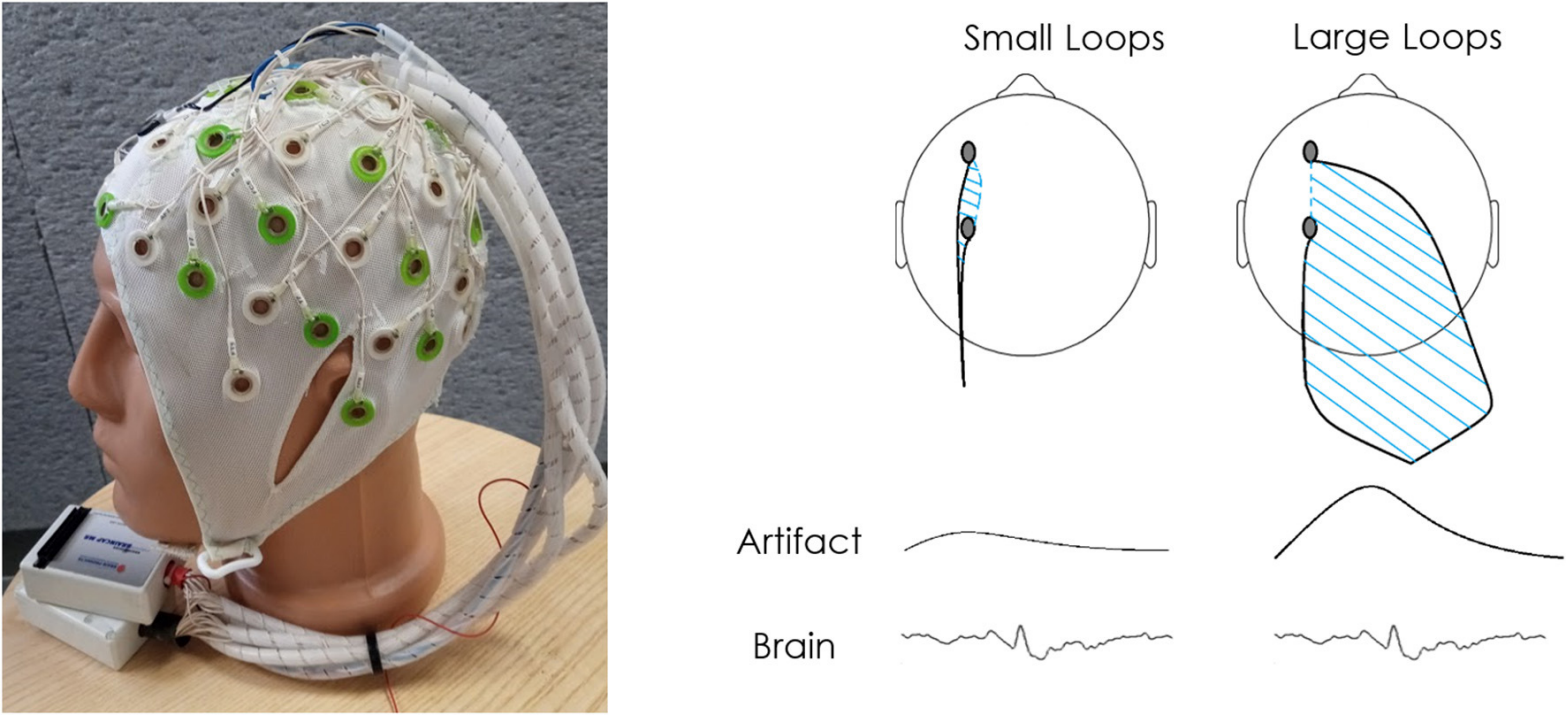
Dual array design and concept. **(A)** The electrode cables are arranged into bundles according to two sets of intersecting lines: longitudinal and transverse. The bundles are grouped into two braids of cables so that the bundles from two neighboring lines travel to the different braids. Each braid is connected to a separate 32 channel referential MR-compatible EEG amplifier. In the dual array arrangement applied in this study, adjacent bundles were sent to a distal amplifier location to cause an increase in the area between electrode cables. A comparison of the bundles is used to differentiate motion artifact from brain signal. **(B)** This wire arrangement is used in order to reduce as much as possible the area within the loop created by two adjacent electrode wires along a single bundle (along bundles) and increase the area within the loop created by adjacent electrodes of different bundles (across bundles). Thus, while the true brain signal should be recorded similarly by adjacent electrodes, motion artifacts should differ depending on the loop created by the bundle each electrode is connected to, allowing separation between signal and noise. For a more detailed explanation of this setup see Klovatch-Podlipsky et al. (32).

All patients underwent EEG-fMRI scans using the dual array EEG (daEEG) method for improved EEG signal artifact identification and removal. EEG was performed using a 64-channel recording system (Figure 1) based on an in-house adaptation of an MR compatible 64-electrode EEG cap (Brain Products GmbH, Gilching, Germany). For a full description of the adapted system see (32).

MRI scans for patients 1–3 *(4)* were performed in a 3.0 T MRI scanner [GE Signa EXCITE *(Siemens Prisma system)*] using a body transmitter coil and an eight *(twenty)* channel head receiver coil. The EEG-fMRI recordings were performed in 20 *(10)* m sessions of scanning; 3–5 *(8)* such sessions were recorded during each patient scan. Patients were instructed to lie still and remain at rest. The helium pump was turned off during the recording as well as air conditioning inside the bore. A T2^*^- weighted, gradient echo, echo planar imaging (EPI) sequence was used for recording the fMRI images (TR/TE/flip angle: 3000/35/90). Thirty-nine *(forty)* axial slices (thickness/gap: 3/0) were collected (FOV: 22 ×22 cm; matrix size: 128 ×128). In addition, a high resolution T1-weighted 3D (1 ×1 ×1 mm) volume was obtained using spoiled gradient echo [SPGR *(MPRAGE)*] sequence.

### EEG Evaluation

EEG data analysis was performed using EEGLAB software (33). Initial EEG processing included gradient interference suppression (FMRIB plug-in for EEGLAB, provided by the University of Oxford Centre for Functional MRI of the Brain), down-sampling from 5,000 to 250 Hz and band-pass filtering to 0.5–40 Hz. After recalculating the data according to the daEEG method, ICA was applied in order to identify and separate components which are differently distributed between the channels. The effect of motion artifacts is larger in data corresponding to measurements between electrodes connected to different bundles in comparison to electrodes connected to the same bundle (Figure 1). The ICA components considered as artifact-affected were removed (32). In order to account for signal changes associated with cardiac activity, the components with high correlation to the recorded ECG trace were removed as well.

Detection of IEDs from the EEG traces after artifact removal was performed manually by a neurologist experienced in EEG interpretation. IEDs of predominant topography and morphology were selected for further analysis. Epochs concurrent with large EEG artifacts were regressed out of the fMRI analysis using the Artifact Detection Tools (ART) toolbox (nitrc.org/projects/artifact_detect/).

### fMRI Analysis

Data analysis was performed using SPM12 software (34). Preprocessing included slice timing correction, 3D motion correction and co-registration to the anatomical image. No normalization to an anatomical atlas was performed. The data was spatially smoothed with an 8 mm full width at half maximum (FWHM) Gaussian kernel. For each session, the first six functional volumes were excluded from analysis. Functional EPI data were automatically aligned and co-registered with 3D anatomical data and manually corrected if necessary. Standard SPM event related fMRI analysis was performed with a general linear model, using the timing of the detected EEG epileptiform waveforms as events. To follow the dynamics of the epileptic events, predictors were modeled with three delays: the standard delay (HRF peak at 5.25 s after the event, corresponding to IED appearance), −3 s delay (HRF peak at 2.25 s after event, corresponding to pre-IED activity) and +3 s delay (HRF peak at 8.25 s after event, corresponding to post-IED activity). In each analysis voxel clusters were significantly active at *p* <0.05 after Family-Wise Error correction (FWE) and a minimum cluster size of 50 adjacent voxels. Earlier peaks showed weaker and more focal activations as expected, thus any cluster of activation composed of over 10 adjacent significant voxels was reported. If no clusters were found significant for standard or early peaks, clusters of >50 adjacent voxels at an uncorrected *p* <0.005 threshold are reported. Sub-threshold clusters within white matter regions were not reported.

## Results

See Supplementary Material for full details of all detected activation clusters (Table A1 in Supplementary Material) and for representative IED traces of each patient (Figure A1 in Supplementary Material).

### Patient 1

**Figure 2:**
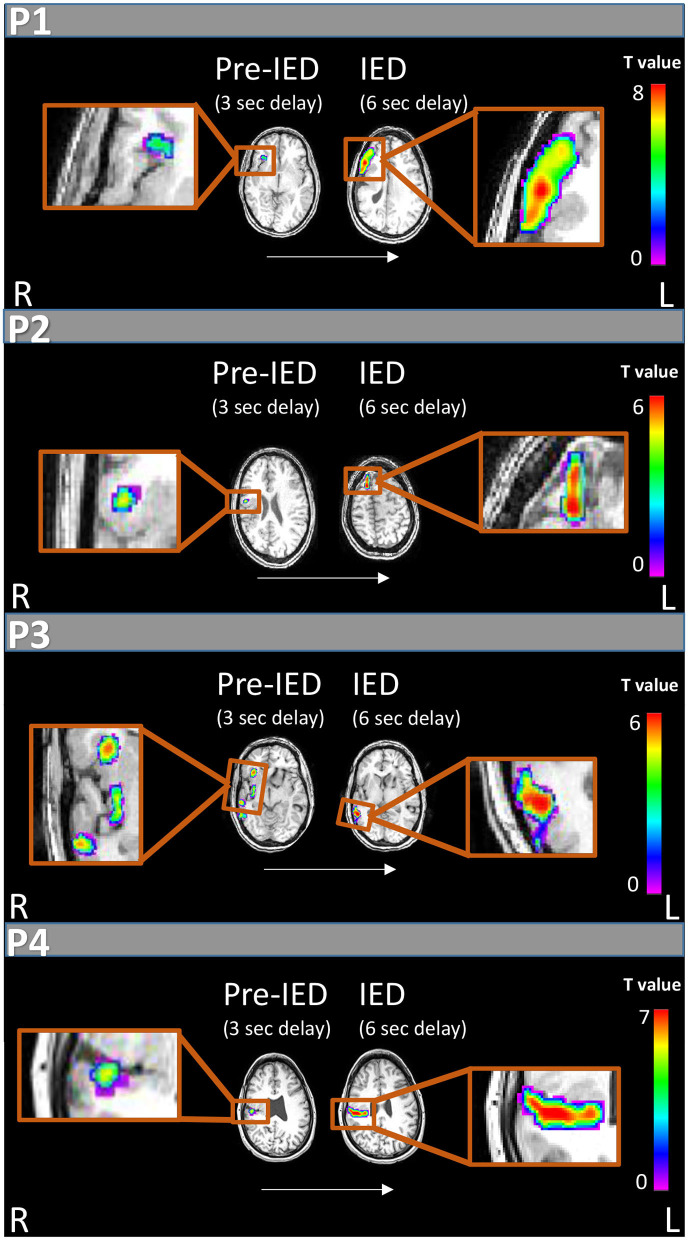
IED and pre-IED correlated fMRI maps for patients 1-4. For each patient areas of peak activation are marked at two time points: 6 s after IED appearance on scalp EEG, showing activity correlated with IEDs according to the standard delay of the HRF peak (right), and 3 s after scalp IED appearance on EEG which shows pre-IED activity (left). All patients show weaker and earlier pre-IED activations within the PMG and later stronger activations on the border of the malformation or outside of it correlated with IED appearance.

During the 40-min recording, 28 spike-and-wave complexes, 11 poly-spike clusters lasting up to 3.5 s and 180 slow waves lasting up to 8 s (1.13 ± 1.36) were detected over right and left fronto-centro-parietal areas. Only spike complexes and clusters were used for the subsequent analyses. Standard delay generated peak activation (*T* = 8.37) at the right inferior frontal gyrus, anterior to the malformation (Figure 2, P1). The FWE corrected BOLD map included the inferior frontal gyrus and insula (areas within the malformation). At −3 s delay the peak activation (*T* = 5.51) was at the right anterior insula (within the malformation, Figure 2, P1) which survived FWE correction. At +3 s delay no significant activation was found, but five clusters of deactivations were observed within the malformation (right pre-central area, right posterior insula) and outside of it in the left temporal operculum, left post central gyrus and left posterior insula all with a peak T-score between 5 and 5.6.

### Patient 2

In the 40-min recording 16 spike-and-wave complexes and 25 poly-spike clusters lasting up to 94 s were detected over right fronto-centro-parietal areas. Standard delay generated peak activation at the right anterior superior frontal gyrus (*T* = 6.2). The related FWE corrected BOLD map included this cluster only (Figure 2, P2). Peak deactivation was found in a bilateral occipital cluster (*T* = 7.0). FWE corrected deactivation maps included additional clusters within the malformation (Table A1 in Supplementary Material). At −3 s delay the peak activation (*T* = 6.3) was at the right superior frontal gyrus and a second cluster in the right parietal operculum cortex within the malformation. The related FWE corrected BOLD map consisted of the described clusters. Two deactivation clusters were found in the right and left occipital lobes. At +3 s delay no significant activation was found, five clusters of deactivations were observed.

### Patient 3

In the 20-min recording 108 spike-and-wave complexes were detected over right centroparietal areas. Standard delay generated peak activation (*T* = 6.21) outside the lesion at the right middle temporal gyrus (Figure 2, P3) along with a superior temporal gyrus activation with similar statistics (*T* = 6.19) and both survived FWE correction. No additional activation or deactivations were found. At −3 s delay the peak activation (*T* = 3.76) was at the right anterior insula. No cluster survived FWE correction, but at *p* = 0.005 without correction this cluster could be observed along with three additional clusters in the right posterior insula, right posterior temporal cortex and a small left occipital cluster. No significant deactivations were found. At +3 s delay no significant activations or deactivations were found.

### Patient 4

In the 60-min recording 62 spike-and-wave complexes were detected over right temporal areas. Standard delay after FWE correction generated peak activation (*T* = 7.9) at the right temporal operculum within a widespread cluster extending to parietal and frontal operculum (Figure 2, P4). Three seconds earlier a peak activation (*T* = 5.61) appeared within a smaller cluster within the temporal operculum. Deactivations correlated with a standard delay were seen in bilateral frontal areas. At +3 s delay one activation was seen at the right post-central gyrus and several deactivation clusters were detected mostly around bilateral frontal areas (Table A1 in Supplementary Material).

## Discussion

The origin of both ictal and interictal activity in PMG has been debated in the literature with findings pointing to an electrophysiological source within the PMG (12) or outside of it (13). In the current study, we set out to evaluate this disagreement by analyzing the simultaneous EEG-fMRI scan results of four patients with unilateral PSPMG, before, during and after the appearance of IED on scalp EEG. All four patients presented BOLD activations within and surrounding the lesion. When applying the HRF with the standard delay, the maximum BOLD activation was found outside or on the borders of the cortical malformation (premotor cortex, superior frontal gyrus, middle temporal gyrus and parietal operculum for patients 1,2,3, and 4, respectively, Figure 2) and corresponded to the maximum BOLD activation overall, considering all evaluated time shifts. Interestingly, we found focal activations correlated with an HRF peak shifted by −3 s in relation to the standard HRF model, and these activations were within the PMG in all four patients (insula in patients 1 and 3, and operculum in patients 2 and 4).

Stutterd and Leventer (35) illustrated that polymicrogyria is highly heterogeneous and the most poorly-delineated among the more common malformations of cortical development. The initial result with the standard HRF seems to support the line of both animal and human studies placing the source of epileptic activity outside of the anatomical malformation. For example, Jacobs et al. (13) proposed that cortical afferents are unable to find appropriate targets within the malformed region and may instead synapse in the adjacent paramicrogyral area, thus suggesting that there is an increase in the number of functional excitatory synapses in the paramicrogyral cortex causing seizures. One previous study evaluated EEG-fMRI in patients with PMG (26) and reported variable maximum BOLD locations: of the 13 analyses showing activations in nine patients, four were inside the lesion, four on the edge and five outside the lesion's boundaries. This discrepancy may stem from the heterogeneity of pathologies evaluated including bilateral, unilateral, frontal and parieto-occipital PMG. The reported variability may have also resulted from the grouping of multiple time delays: at each voxel, the maximum t value was taken from four t-maps created using four hemodynamic response functions with peaks at 3, 5, 7, and 9 s. Such an analysis has the potential to obscure earlier and possibly weaker activations.

In this work, we thus concentrated on a homogenous group of patients with unilateral PSPMG. Indeed, we found focal activations in the analysis correlated with an HRF peak shifted by −3 s in relation to the standard HRF model, and these activations were within the PMG in all four patients (insula in patients 1 and 3, and operculum in patients 2 and 4). On this basis, we propose that IEDs in PSPMG initiate in a relatively small cortical structure within the lesion itself. The stronger activations observed with the standard delay are suggested to represent the shift and expansion of the interictal generators to adjacent, more organized or superficial cortical structures, as these structures are more capable of producing currents detected on the scalp. BOLD responses preceding interictal activity have been previously observed and discussed (36–39). These studies point to the significance of such early, often weaker BOLD responses and their potential clinical relevance. Jacobs et al. (36) suggested that early hemodynamic activity seen in the spike field prior to scalp EEG result from neuronal activity invisible to scalp EEG which is more focal and which systematically leads to a more widespread response. This early activity has also been associated with other metabolic events preceding the IED and suggested to be associated with its generation (36, 38).

Several models have been proposed for the epileptogenicity of the PMG cortex. Takano (12) found reduced parvalbumin-immunoreactive interneurons within the medial parts of the PMG compared to more lateral parts. Stouffer et al. (40) report malfunction of the cytoskeleton caused by mutations of neural migration genes leading to an excitatory-inhibitory imbalance. Our study does not allow the evaluation of such hypotheses and further research, primarily with animal models, is necessary to improve our understanding of these mechanisms. Moreover, our study only examines IED related activity, which may be governed by a divergent neuro-electrical circuit as compared to that which governs ictal phenomena (41). Nevertheless, this result implies several mechanistic traits of the processes underlying epileptic seizures secondary to PSPMG. From the spread of both activations and deactivations we observed (with the standard delay and +3 s delays), it appears that a complex interaction of network nodes within the PMG and outside it is involved during IEDs. Such a heterogeneous network of cortical activation has also been reported in human intracranial studies. Ramantani (42) found a network of interictal and ictal activity including the PMG and medial temporal cortex and Chassoux et al. (14) reported epileptogenicity in the PMG cortex extending beyond the visible abnormality.

Our work further suggests that the focal activity preceding later widespread interictal events may be overlooked using standard EEG-fMRI. Such activity may also be undetected with iEEG as shown in one study which explored the area of early BOLD activation with depth electrodes. In this study the early focal activity was correlated with interictal events on iEEG in only one of four patients examined (38). The authors suggest this finding may point to the early BOLD activations reflecting non-synchronized neuronal activity or non-neuronal mechanisms. In addition, while intracranial recordings offer an opportunity to directly record local fields, they have the disadvantage of under-sampling relevant areas. As resective surgery was not performed on these patients, this study cannot verify whether the reported initial areas of activation indeed identify the epileptogenic zone. Further studies using intracranial recordings and clinical outcomes of surgery are needed to address this question. Notwithstanding, these results support the exploration of interictal dynamics at the timescale of BOLD activity for inferring patterns of epileptic spread from the source areas.

When considering the presented results and their interpretation it is important to note several limitations of the applied methodology. Primarily, the hemodynamic response itself may vary within and between patients, reflecting differences in the BOLD signal among brain areas and conditions, as suggested by previous studies (43, 44). Such differences could also account for the activity associated with the different HRF peaks, perhaps pointing to sources of several interictal event types and not the shift of a specific IED source. In addition, the averaged IEDs may themselves carry information regarding the location of earlier activity as suggested in studies which use high resolution source imaging with MEG or EEG data (1, 18). In future studies it may thus be interesting to compare the sources of averaged IEDs recorded by EEG or MEG to the hemodynamic activations seen with fMRI. In these cases, the appearance of early focal activation clusters within the PMG may still be indicative of relevant treatment targets as previously discussed. Our finding of preceding activations within the PSPMG support the notion that IEDs initiate inside the PMG and propagate to neighboring cortex. As well as improving our understanding of epilepsy related processes in PMG, this finding has the potential to assist in tailoring invasive treatments: from directing depth electrode implantation, to guiding resection and neuro-stimulation probes. Finally, this finding supports the use of EEG-fMRI for tracing IED dynamics with potential benefits for improved localization of epileptogenic areas.

## Data Availability Statement

The raw data supporting the conclusions of this article will be made available by the authors, without undue reservation.

## Ethics Statement

The studies involving human participants were reviewed and approved by Tel-Aviv Sourasky Medical Center Ethical Review Board. The patients/participants provided their written informed consent to participate in this study. Written informed consent was obtained from the individual(s) for the publication of any potentially identifiable images or data included in this article.

## Author Contributions

NC, TG, and YE carried out data analysis and wrote the manuscript. TG suggested the project. MM, FF, TH, NC, and TG supervised the work. OA, MM, and FF provided clinical and diagnostic information. FF, TG, OA, TH, and GG revised the manuscript. All authors contributed to the article and approved the submitted version.

## Conflict of Interest

FF, TH, and MM are authors of patent: Device for use in electro-biological signal measurement in the presence of a magnetic field, WO2012046237, 12.04.2012. The remaining authors declare that the research was conducted in the absence of any commercial or financial relationships that could be construed as a potential conflict of interest.
