# Supplementary material for: Interictal Epileptiform Discharge Dynamics in Peri-sylvian Polymicrogyria Using EEG-fMRI
Source: Front Neurol. 2021 Jun 3;12:658239. doi: 10.3389/fneur.2021.658239 (PMC8212705; doi:10.3389/fneur.2021.658239)
Supplement: Supplementary file 1 [file Data_Sheet_1.pdf]

Appendix A:
Supplementary Material

Figure A.1. Traces of representative IEDs used for the event-related fMRI analysis of patients 1-4.

P1

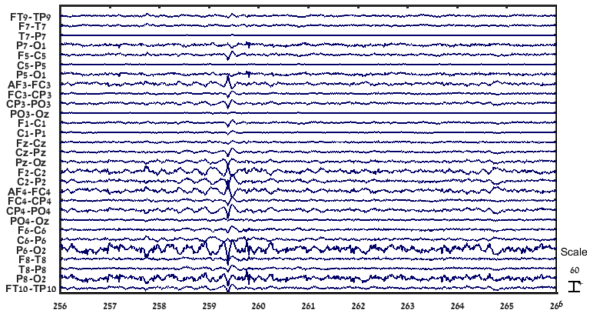

P2

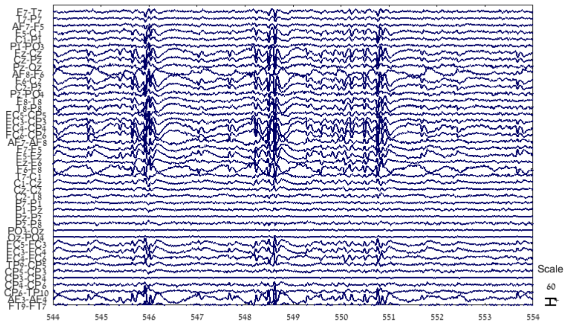

P3

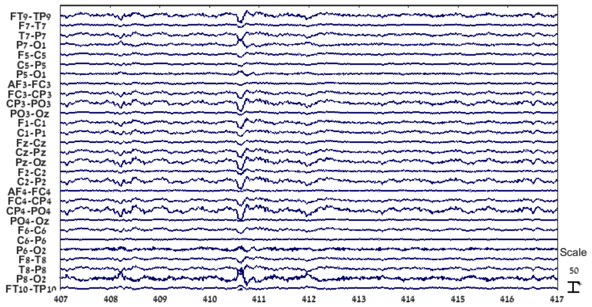

P4

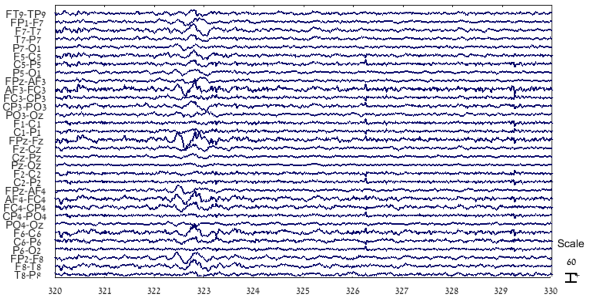

Table A.1. Location and statistical properties of significant clusters of activation and deactivation for each of the four patients included in the study. For each patient significant activations are reported for each of three time shifts in relation to the appearance of IEDs on the scalp, such that a 5.25 second shift represents the time shift used for standard BOLD analyses, a -3 second shift (2.25 seconds after IED appearance) corresponds to pre-IED activity, and a +3 second shift (8.25 seconds after IED appearance) to post-IED activity. Clusters were considered significant if they survived family wise error (FWE) correction at p<0.05. If no cluster survived, significant clusters at an uncorrected threshold of p<0.001 are reported. R, right; L, left; FWE, Family wise error; da, deactivation; unc, uncorrected.

Patient	Delay relative to standard HRF (sec)	Location	Cluster p(FWE-corrected)	Cluster equivk	Cluster p(unc)	Peak p(FWE-corrected)	Peak T	Peak equivZ	Peak p(unc)
**P1**	**-3**	L anterior insula	1.82E-03	21	3.56E-02	3.09E-03	5.291	5.240	8.01E-08
		L cerebellum	5.14E-03	12	1.01E-01	7.55E-03	5.098	5.052	2.18E-07
	**0**	R inferior frontal gyrus	0.00E+00	1105	1.04E-21	1.28E-11	8.371	65535	4.44E-16
		R cingulate cortex	2.20E-07	143	4.28E-06	1.57E-06	6.707	6.606	1.98E-11
		L cerebellum	5.65E-13	417	1.10E-11	3.43E-06	6.577	6.481	4.56E-11
		L anterior insula	2.05E-05	72	4.00E-04	4.46E-06	6.532	6.438	6.06E-11
		R precentral gyrus	1.24E-06	114	2.42E-05	1.14E-05	6.370	6.283	1.66E-10
		L posterior corpus collosum	7.51E-08	162	1.46E-06	8.81E-04	5.558	5.499	1.91E-08
		R superior frontal gyrus	3.71E-04	36	7.24E-03	2.57E-03	5.339	5.286	6.24E-08
		R posterior corpus collosum	6.27E-03	10	1.23E-01	7.07E-03	5.121	5.075	1.94E-07
		R caudate	5.47E-03	11	1.07E-01	1.02E-02	5.039	4.995	2.94E-07
		R cerebellum	5.47E-03	11	1.07E-01	1.32E-02	4.981	4.938	3.95E-07
	**+3 (da)**	L superior temporal gyrus	6.47E-06	91	1.26E-04	7.54E-04	5.585	5.525	1.65E-08
		L post-central gyrus	4.09E-13	437	7.97E-12	8.49E-04	5.561	5.502	1.88E-08
		R (lesion border)	6.16E-07	129	1.20E-05	1.04E-03	5.519	5.462	2.36E-08
		L temporal operculum	3.93E-05	65	7.66E-04	2.25E-03	5.362	5.309	5.52E-08
		R temporal operculum	1.06E-04	52	2.06E-03	4.44E-03	5.218	5.169	1.18E-07
**P2**	**-3**	R superior frontal gyrus	4.27E-05	67	8.32E-04	1.24E-05	6.338	6.251	2.04E-10
		R parietal operculum	3.34E-03	16	6.52E-02	4.47E-03	5.199	5.150	1.30E-07
	**-3 (da)**	R occipital pole	1.47E-06	120	2.87E-05	3.59E-04	5.716	5.652	7.95E-09
		L lingual gyrus	2.19E-09	248	4.27E-08	1.48E-03	5.433	5.377	3.79E-08
	**0**	R superior frontal gyrus	0	81	0	0	6.200	6.120	0
	**0 (da)**	Bi occipital lobe	0	2760	1.59E-37	2.33E-07	7.002	6.886	2.87E-12
		L postcentral gyrus	0	802	7.17E-17	4.39E-05	6.112	6.034	8.00E-10

		R parietal operculum	6.32E-10	275	1.23E-08	2.78E-04	5.766	5.700	5.99E-09
		L anterior paracentral gyrus	1.27E-04	52	2.48E-03	9.77E-04	5.517	5.459	2.39E-08
		L paracentral lobule	7.92E-06	92	1.54E-04	1.08E-03	5.498	5.440	2.66E-08
	**+3 (da)**	R occipital lobe	7.77E-16	637	1.49E-14	2.65E-05	6.203	6.121	4.63E-10
		L lingual gyrus	4.85E-06	100	9.46E-05	7.68E-05	6.009	5.935	1.47E-09
		R middle frontal gyrus	1.75E-05	80	3.41E-04	1.05E-03	5.503	5.445	2.58E-08
		L cerebellum	2.80E-05	73	5.46E-04	1.56E-03	5.422	5.366	4.02E-08
		L occipital pole	7.57E-05	59	1.48E-03	4.45E-03	5.200	5.151	1.29E-07
**P3**	**-3**	R anterior insula **n.s.**	6.59E-01	129	4.30E-02	7.55E-01	3.760	3.720	0
		R middle temporal gyrus **n.s.**	1.46E-01	263	6.00E-03	7.95E-01	3.720	3.690	0
		R posterior insula **n.s.**	9.36E-01	76	1.09E-01	8.89E-01	3.610	3.580	0
		L angular gyrus **n.s.**	9.43E-01	74	1.13E-01	9.88E-01	3.360	3.340	0
	**0**	R middle temporal gyrus	0	172	0	0	6.210	6.050	0
		R supramarginal gyrus	0	55	2.00E-03	0	6.190	6.040	0
**P4**	**-3**	R temporal operculum	1.21E-05	23	2.36E-04	1.10E-03	5.614	5.574	1.24E-08
		R angular gyrus	1.27E-04	14	2.48E-03	2.58E-03	5.461	5.424	2.91E-08
	**0**	R temporal operculum	0	536	3.21E-30	1.47E-10	7.995	Inf	1.89E-15
		L cerebellum	1.83E-12	113	3.57E-11	1.43E-06	6.704	6.636	1.61E-11
	**0 (da)**	R posterior cingulate gyrus	5.42E-11	90	1.06E-09	6.57E-08	7.158	7.076	7.42E-13
		L frontal operculum	1.59E-12	114	3.10E-11	1.25E-06	6.723	6.655	1.42E-11
		L precental gyrus	2.95E-11	94	5.75E-10	7.62E-06	6.445	6.384	8.61E-11
		R frontal operculum	8.63E-09	59	1.68E-07	4.72E-04	5.763	5.720	5.34E-09
	**+3**	R post-central sulcus	0	213	1.11E-16	7.08E-10	7.785	7.676	8.22E-15
	**+3 (da)**	R frontal operculum	0	2217	8.60E-77	0	8.795	Inf	4.44E-16
		L superior insula	0	1607	4.18E-62	0	8.678	Inf	4.44E-16
		R lingual gyrus	0	5018	7.44E-132	0	8.659	Inf	4.44E-16
		R lateral occipito-temporal gyrus	2.14E-09	67	4.17E-08	9.73E-10	7.741	7.636	1.12E-14
		L thalamus	1.35E-12	115	2.63E-11	1.19E-08	7.399	7.309	1.35E-13
		R precentral gyrus	1.84E-11	97	3.59E-10	6.63E-06	6.467	6.405	7.49E-11
		L inferior frontal sulcus	4.42E-13	123	8.61E-12	1.51E-04	5.958	5.910	1.71E-09
		L superior frontal gyrus	1.45E-08	56	2.83E-07	1.82E-04	5.927	5.879	2.06E-09
